# Introduction of Medical Emergency Teams in Australia and New Zealand: a multi-centre study

**DOI:** 10.1186/cc6857

**Published:** 2008-04-07

**Authors:** Daryl Jones, Carol George, Graeme K Hart, Rinaldo Bellomo, Jacqueline Martin

**Affiliations:** 1 Australian and New Zealand Intensive Care Research Centre, Department of Epidemiology and Preventive Medicine, Monash University, 89 Commercial Road, Melbourne 3004, Victoria, Australia; 2 Australian and New Zealand Intensive Care Society Adult Patient Database, 10 Ievers St, Carlton, Melbourne, Victoria 3053, Australia; 3 Intensive Care Research and Staff Specialist Intensive Care, Austin Hospital, Studley Rd, Heidelberg, Melbourne, Victoria 3084, Australia; 4 Australian and New Zealand Intensive Care Society Research Centre for Critical Care Resources, 10 Ievers St, Carlton, Melbourne, Victoria 3053, Australia

## Abstract

**Introduction:**

Information about Medical Emergency Teams (METs) in Australia and New Zealand (ANZ) is limited to local studies and a cluster randomised controlled trial (the Medical Emergency Response and Intervention Trial [MERIT]). Thus, we sought to describe the timing of the introduction of METs into ANZ hospitals relative to relevant publications and to assess changes in the incidence and rate of intensive care unit (ICU) admissions due to a ward cardiac arrest (CA) and ICU readmissions.

**Methods:**

We used the Australian and New Zealand Intensive Care Society database to obtain the study data. We related MET introduction to publications about adverse events and MET services. We compared the incidence and rate of readmissions and admitted CAs from wards before and after the introduction of an MET. Finally, we identified hospitals without an MET system which had contributed to the database for at least two years from 2002 to 2005 and measured the incidence of adverse events from the first year of contribution to the second.

**Results:**

The MET status was known for 131 of the 172 (76.2%) hospitals that did not participate in the MERIT study. Among these hospitals, 110 (64.1%) had introduced an MET service by 2005. In the 79 hospitals in which the MET commencement date was known, 75% had introduced an MET by May 2002. Of the 110 hospitals in which an MET service was introduced, 24 (21.8%) contributed continuous data in the year before and after the known commencement date. In these hospitals, the mean incidence of CAs admitted to the ICU from the wards changed from 6.33 per year before to 5.04 per year in the year after the MET service began (difference of 1.29 per year, 95% confidence interval [CI] -0.09 to 2.67; *P *= 0.0244). The incidence of ICU readmissions and the mortality for both ICU-admitted CAs from wards and ICU readmissions did not change. Data were available to calculate the change in ICU admissions due to ward CAs for 16 of 62 (25.8%) hospitals without an MET system. In these hospitals, admissions to the ICU after a ward CA decreased from 5.0 per year in the first year of data contribution to 4.2 per year in the following year (difference of 0.8 per year, 95% CI -0.81 to 3.49; *P *= 0.3).

**Conclusion:**

Approximately 60% of hospitals in ANZ with an ICU report having an MET service. Most introduced the MET service early and in association with literature related to adverse events. Although available in only a quarter of hospitals, temporal trends suggest an overall decrease in the incidence of ward CAs admitted to the ICU in MET as well as non-MET hospitals.

## Introduction

Rapid Response Systems (RRSs) have been introduced into hospitals to identify and treat at-risk ward patients in an attempt to reduce unplanned intensive care unit (ICU) admissions and cardiac arrests (CAs) [1-3]. In Australia and New Zealand (ANZ), the most common form of RRS is the ICU-based Medical Emergency Team (MET) system, first described by Lee and colleagues in 1995 [4]. METs have been shown to reduce the incidence of in-hospital CAs in a number of single-centre before-and-after studies [5-9]. A recently published cluster randomised controlled trial (the Medical Emergency Response and Intervention Trial [MERIT] [10]) involving 23 Australian hospitals, however, did not confirm this finding.

In the United States, RRSs have been introduced into multiple hospitals in response to the '5 Million Lives campaign' promoted by the Institute of Health Improvement (IHI) [11]. On the other hand, the degree of uptake and factors affecting the introduction of MET services into hospitals in ANZ are not well described. Similarly, no aggregate information exists on how the introduction of MET systems might have affected relevant outcome measures outside the setting of the cluster randomised trial, a comparative study of three hospitals, or single-centre before-and-after studies.

The Australian and New Zealand Intensive Care Society (ANZICS) Research Centre for Critical Care Resources (ARCCCR) maintains a database recording information on critical care resources, including the timing of introduction of METs. In addition, ANZICS maintains an Adult Patient Database (ANZICS-APD), which currently contains information on the demographics, admissions source, and outcomes of more than 450,000 ICU admissions. The development and details of the ANZICS-APD have been described in detail elsewhere [12].

The aims of this study were (a) to describe the timing and extent of the introduction of MET services into ANZ hospitals in relation to relevant publications, (b) to assess the association between MET service introduction and the incidence and rate of ICU admissions due to ward CAs, (c) to assess the association between MET service introduction and the incidence and rate of ICU readmissions, and (d) to assess changes in the same adverse events in hospitals that had not introduced an MET service.

## Materials and methods

### Ethical considerations

The collection, analysis, and reporting of de-identified data by the ANZICS-APD comply with Australian Commonwealth legislation (1994) enabling national quality assurance activities. They also comply with the quality assurance amendment of the Australian Health Insurance Act (1973) [12]. This enables ethical approval for research projects to be undertaken using the information contained within the database.

### Assessment on timing of introduction of MET service

We obtained information from a database maintained by the ANZICS ARCCCR and derived from surveys of ICU resources and activity. The information related to the timing of commencement of an MET into hospitals in ANZ which were not involved in the MERIT study [10]. Hospitals in this database are characterised by the presence of an ICU and were categorised into 'MET: commencement date known', 'MET: commencement date unknown', 'No MET service', or 'MET status unknown'. Graphs were constructed to display the cumulative uptake of METs with time between the period from February 1995 to May 2005. The timing of commencement was assessed for hospitals overall and separately for 'metropolitan', 'private', 'rural/regional', and 'tertiary' hospitals as classified in the ARCCCR database.

### MET service commencement in relation to publications

An electronic search was conducted to identify literature related to serious adverse events and METs to assess the timing of MET introduction in relation to such literature. Studies were selected from a Medline search from 1990 to 2006 using the key words 'adverse event', 'medical emergency team', 'cardiac arrest', and 'rapid response team'. The date of these publications was then related to the timing of commencement of MET services.

### Assessment of the effect of MET service commencement on adverse events

The ANZICS-APD was interrogated to obtain data on the incidence of ICU admissions secondary to CAs in ward patients and on the incidence of readmissions to the ICU.

### Hospital eligibility criteria

Hospitals were eligible for analysis if they had an MET service with a known commencement date and had contributed to the ANZICS-APD for the two continuous years spanning the introduction of the MET service (12 months before and 12 months after). Hospitals were excluded from analysis if they were participants in the MERIT study [10], if the MET status or time of MET commencement was unknown, or if they had no events recorded at baseline.

#### Definition of adverse events and data extraction from ANZICS-APD

The APD was interrogated using commercially available software (SAS for Windows; SAS Institute Inc., Cary, NC, USA) for data in the 12 months before and 12 months after commencement of the MET service. In the case of ward CAs, the patient cohort was constructed by restricting the 'ICU admission source' field to 'patients admitted from the ward' and restricting the 'admission diagnostic codes' field to the APACHE (Acute Physiology and Chronic Health Evaluation) III 'non-operative diagnostic code 114 – post cardiac arrest'. The cohort of patients experiencing ICU readmission was constructed by including all patients admitted to the ICU on two or more occasions in the same hospital admission, regardless of admissions source. We also obtained information on the overall number of ICU admissions and the hospital mortality of patients admitted after a ward CA or readmission.

We assessed similar changes in hospitals that had contributed at least 24 months of data to the APD during the same period (2000 to 2005) but had not introduced an MET service and had not participated in the MERIT study by comparing the first year of data submission to the second. Finally, in an additional sensitivity assessment, we extended our analysis to hospitals involved in the MERIT study which had submitted information to the APD before participation in the MERIT study and which had continued to submit data thereafter.

### Data analysis and statistics

Descriptive data are presented as raw numbers and as a percentage of overall cases or events. Data on adverse events (ICU admission due to ward CA and readmission to ICU) are presented as means ± standard deviation for absolute values and rates of events (adjusted for total ICU admissions) in the 12-month periods before and after the commencement of the MET service.

The difference in the incidence and hospital mortality for adverse events before and after commencement of the MET was tested for with the Wilcoxon signed rank test. A similar comparison was performed for hospitals that had not introduced an MET service using the first year of data as baseline and the second year as comparator. Finally, an additional and similar analysis was performed for hospitals that had participated in the MERIT study. A *P *value of less than 0.05 was considered statistically significant.

## Results

### MET service status in 'non-MERIT' ANZ hospitals

**Figure 1:**
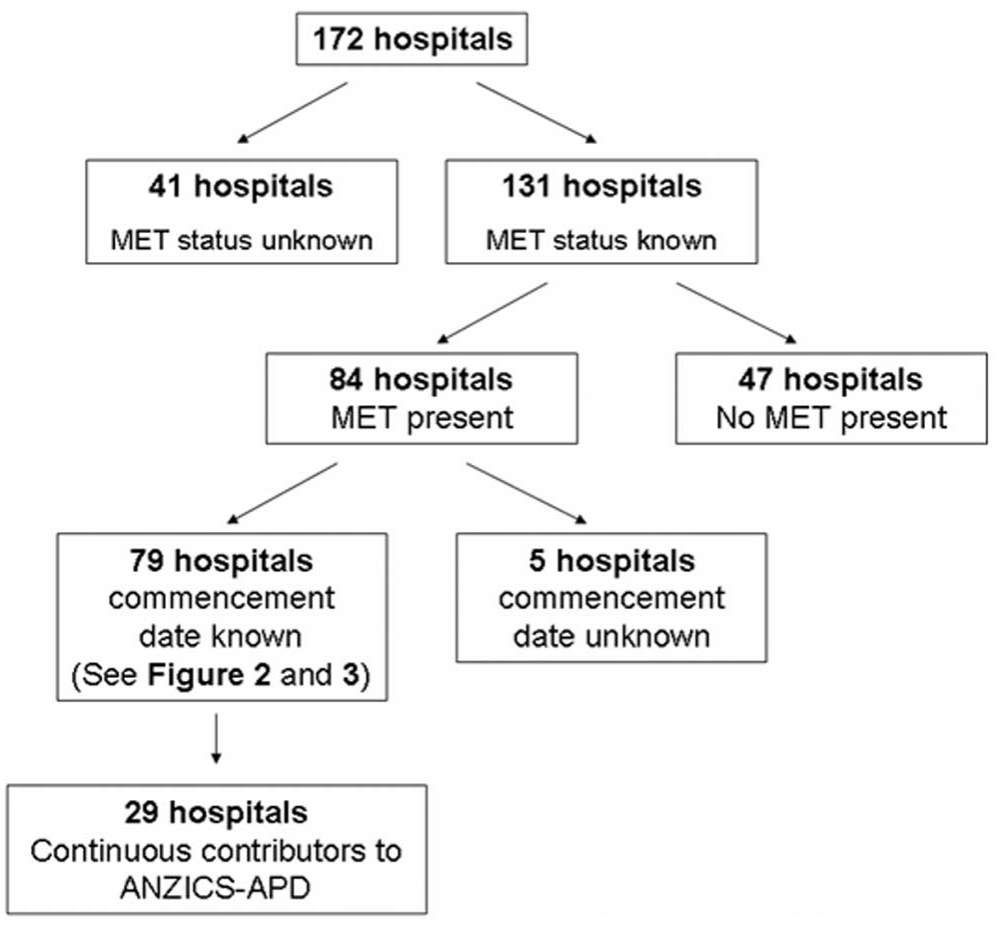
Flow diagram of the Medical Emergency Team (MET) status of 172 hospitals in Australian and New Zealand with intensive care units. The diagram does not include hospitals participating in the Medical Emergency Response and Intervention Trial. ANZICS-APD, Australian and New Zealand Intensive Care Society Adult Patient Database.

The MET status was known for 131 of the 172 (76.2%) ANZ hospitals that did not participate in the MERIT study (Table 1). The proportion of cases in which the MET status was known varied from 66.7% (private hospitals) to 96% (tertiary hospitals) depending on hospital category. In 94% of hospitals with an MET service, the commencement date was known (Figure 1, Table 1).

**Table 1:** Medical Emergency Team (MET) service status in 172 hospitals in Australia and New Zealand

Hospital category	MET service commencement date known	MET service commencement date unknown	No MET service	MET status unknown, number (percentage of total^a^)	Total
All hospitals	79	5	47	41 (23.8)	172
Metropolitan	18	1	13	3 (8.6)	35
Private	28	1	11	20 (33.3)	60
Regional	18	3	14	17 (32.7)	52
Tertiary	15	0	9	1 (4)	25

Hospitals participating in the Medical Emergency Response and Intervention Trial are not included in the data above. ^a^'Total' refers to the total number of hospitals in each hospital category.

In the 131 'non-MERIT' hospitals in which the MET status was known, 64.1% of hospitals stated that an MET service had been introduced (Figure 1, Table 2). In these hospitals, the proportion of hospitals with an MET service varied from 62.5% (regional) to 72.5% (private) depending on hospital category (Table 2).

**Table 2:** Proportion with and without a Medical Emergency Team (MET) amongst hospitals with information on MET status

Hospital category	MET service present	No MET service	Percentage with MET service^a^
All hospitals	84	47	64.1
Metropolitan	19	13	59.4
Private	29	11	72.5
Regional	21	14	62.5
Tertiary	15	9	64.1

^a^Indicates the percentage with MET service only for 131 'non-MERIT' hospitals in which the MET status of the hospital is known. MERIT, Medical Emergency Response and Intervention Trial.

### Timing of MET service commencement in relation to publications

**Figure 2:**
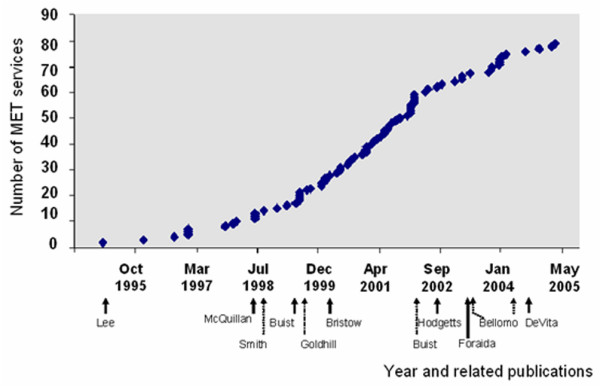
Uptake of Medical Emergency Team (MET) services into those hospitals in Australia and New Zealand for which the MET status is known. Each data point represents the cumulative total of MET services commenced (y-axis) at the corresponding time (x-axis). The commencement of the MET service at Liverpool Hospital (University of New South Wales, Sydney, Australia) (June 1989) is omitted for the purpose of presentation. Shown below the x-axis are the first authors of publications related to adverse events and METs: Lee, *et al*. [4]; McQuillan, *etal*. [16]; Smith and Wood [17]; Buist, *et al*. [14]; Goldhill, *et al*. [15]; Bristow, *et al*. [13]; Buist, *et al*. [6]; Hodgetts [21]; Foraida [22]; Bellomo, *et al*. [5]; and DeVita [7].

**Figure 3:**
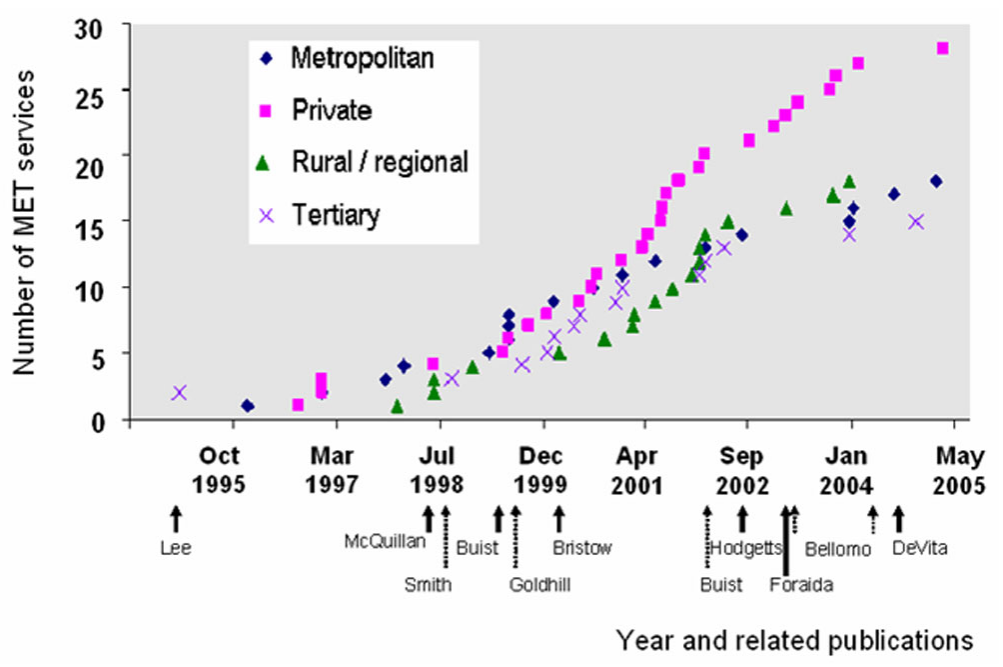
Uptake of Medical Emergency Team (MET) services into various categories of hospitals in Australia and New Zealand for which the MET status is known. Each data point represents the cumulative total of the number of MET services commenced (y-axis) at the corresponding time (x-axis). The commencement of the MET service at Liverpool Hospital (University of New South Wales, Sydney, Australia) (June 1989) is omitted for the purpose of presentation. Shown below the x-axis are the first authors of publications related to adverse events and METs: Lee, *et al*. [4]; McQuillan, *et al*. [16]; Smith and Wood [17]; Buist, *et al*. [14]; Goldhill, *et al*. [8]; Bristow, *et al*. [13]; Buist, *et al*. [6]; Hodgetts [21]; Foraida [22]; Bellomo, *et al*. [5]; and DeVita [7].

In the 79 hospitals in which the MET commencement date was known, 75% of MET services had commenced by May 2002 (Figure 2). A similar pattern of uptake was seen for all hospital categories (Figure 3). Prior to May 2002, there were three publications related to the MET and several publications describing antecedents to serious adverse events in hospital patients [13-18].

### Effect of MET service commencement on adverse events

Of the 79 hospitals in which the MET service commencement date was known, 29 had also contributed continuous data to the ANZICS-APD in the year before and after the date of MET service introduction (Figure 1). In these 29 hospitals, sufficient data on CAs were available in 24. In these 24 hospitals, there was a statistically significant reduction (*P *= 0.0244) in the incidence of ward CAs admitted to the ICU in the year after the introduction of an MET service. A similar decrease was seen in their rate (events per 1,000 admissions) (Table 3).

**Table 3:** Number and frequency of ICU admissions due to cardiac arrest and ICU readmissions

Outcome measure	Year before MET introduction, mean (SD)	Year after MET introduction, mean (SD)	*P *value
Cardiac arrests admitted from ward to ICU per year	6.33 (5.29)	5.04 (5.52)	0.024
Rate of admitted ward cardiac arrests (events per 1,000 ICU admissions)	8.66 (4.45)	6.13 (4.70)	0.016
ICU readmissions per year following ICU discharge	28.22 (32.05)	29.18 (36)	0.91
ICU readmission rate (events per 1,000 ICU admissions)	36.00 (19.08)	34.98 (21.68)	0.74

ICU, intensive care unit; MET, Medical Emergency Team; SD, standard deviation.

Data from 29 hospitals in Australia and New Zealand for 12 months before and 12 months after MET service introduction.

The rates of survival to hospital discharge for patients admitted to the ICU after a ward CA were 37.9% before the introduction of the MET and 38.3% after the introduction of the MET (*P *= 0.779) (Table 4). There was no statistically significant reduction in the incidence of ICU readmissions (Table 3) or hospital survival of ICU readmissions in association with the introduction of the MET service into the hospitals studied (Table 4).

**Table 4:** Hospital mortality of patients requiring ICU admission due to ward cardiac arrest and ICU readmission*

Outcome measure	Percentage survival in the year before MET introduction, mean (SD)	Percentage survival in the year after MET introduction, mean (SD)	*P *value
Cardiac arrests admitted from ward to ICU	37.9 (30.6)	38.3 (27.0)	0.779
ICU readmissions following ICU discharge	79.4 (16.2)	77.5 (20.8)	0.808

ICU, intensive care unit; MET, Medical Emergency Team; SD, standard deviation.

*Data from the 12 months before and after introduction of an MET service into 29 hospitals in Australia and New Zealand

### Adverse events in hospitals without an MET service

We identified 47 hospitals with no MET service (Figure 1). Of these, 16 had contributed data for two years during the period from 2002 to 2005 and did not participate in the MERIT study: 4 private hospitals, 6 metropolitan hospitals, 2 regional hospitals, and 4 tertiary hospitals. In these hospitals, data were obtained for the years 2002 to 2005. When comparing the first year with sufficient data to the following year, we found a decrease in the incidence of CAs from 5 to 4.2 per year (*P *= 0.3). Similar to MET hospitals, there was no change in other outcome measures (Table 5).

**Table 5:** Characteristics of ICU admissions due to cardiac arrest and ICU readmission service*

Outcome measure	First year (baseline), mean (SD)	Second year (follow-up), mean (SD)	*P *value
Cardiac arrests admitted from ward to ICU per year	5.0 (4.3)	4.2 (3.9)	0.33
Rate of admitted ward cardiac arrests (events per 1,000 ICU admissions)	9.0 (6.1)	7.1 (7.1)	0.30
ICU readmissions per year following ICU discharge	23.2 (22.1)	28.2 (28.1)	0.08
ICU readmission rate (events per 1,000 ICU admission)	36.1 (19.2)	37.2 (19.1)	0.99
Outcomes	Percentage survival in first year	Percentage survival in second year	*P *value
Cardiac arrests	29.4 (28.3)	38.8 (24.9)	0.35
ICU readmissions	87.3 (13.9)	88.4 (8.1)	0.77

ICU, intensive care unit; MET, Medical Emergency Team; SD, standard deviation.

*Data from the baseline 12 months and follow-up 12 months in 16 hospitals that had not introduced an MET service

### MERIT hospitals

Twenty-three hospitals participated in the MERIT study. Of those randomly assigned to an MET service (n = 12), all continued to have an MET system in 2007. Of those randomly assigned to the control arm (n = 11), five had introduced an MET service by 2005. Twelve hospitals could be identified which participated in MERIT, had an MET system, contributed to the database, and had contributed data for at least one year before the introduction of the MET and one year thereafter. Six hospitals could be identified which participated in MERIT, did not have an MET system, contributed to the database, and had contributed data for at least two consecutive years during our study period. These hospitals showed no temporal trends in readmission rates. However, both control hospitals and MET hospitals showed a trend toward a decreased percentage of ICU admissions being secondary to CAs (*P *= 0.11 and *P *= 0.1, respectively). When hospitals were analysed in their aggregate, this temporal trend was statistically significant (*P *= 0.023).

## Discussion

### Summary of study findings

We studied the introduction of MET services into 172 hospitals in ANZ which did not participate in the MERIT study [10] and assessed the association between this introduction and the pattern of adverse events. We similarly and separately also assessed hospitals from the MERIT study. Information on MET status was available in more than three quarters of hospitals and approximately 60% of these had introduced an MET service. Most hospitals introduced MET services following publications related to adverse events rather than after studies reporting the effectiveness of the MET. In hospitals (n = 24) for which information was available, the incidence of CAs was lower in the year after the introduction of the MET service compared with the year before its introduction. No changes were seen in other outcome measures. Similar changes were found in a cohort of hospitals that had not introduced an MET service and in hospitals that participated in the MERIT study.

### Timing of introduction of MET services

In the United States, the IHI emphasised that RRSs were an integral part of the 100,000 Lives Campaign, which commenced in January 2005 [19,20]. They subsequently reported that 100 hospitals have implemented an RRS and that more than half of the 2,500 hospitals that joined the campaign said they intended to follow suit [19]. In the present study of hospitals in ANZ, most MET services were introduced before May 2002. Prior to this date, only one publication [6] reported a reduction in the incidence of unexpected CAs in association with the introduction of an MET service. Other studies of the MET published in this period either described the MET as a concept [1] or failed to show a reduction in CAs in association with MET service introduction [13]. These findings suggest that most hospitals that have introduced an MET service did so primarily in response to presentations by opinion leaders or to studies describing antecedents to unexpected CAs and unplanned ICU admissions.

### Effect of MET service introduction on adverse events

Our study identified that the introduction of an MET service was associated with a significant reduction in the incidence and rate of ICU admissions due to a ward CA. However, this effect could be measured in only 24 of the 84 hospitals with an MET service. We are unable to comment on changes in the incidence of CAs in hospitals that did not fulfil these criteria or where the MET status was unknown. In a small and unmatched cohort of hospitals (n = 16) without an MET which contributed 24 months of consecutive data during the same time frame, however, similar changes in outcome were seen. Finally, we also obtained information on those hospitals that had participated in the MERIT study and had contributed sufficient data for analysis. We found that 5 of 11 MERIT control hospitals had introduced an MET system and that the temporal trends toward reduced CA admission to the ICU seen in the main cohort were confirmed in MERIT hospitals.

### Study strengths and limitations

To our knowledge, this is the only study to assess the implementation of METs in two countries and the timing of such implementation. It is also the first to seek to relate the introduction of METs to available evidence. It is also the first multi-centre before-and-after comparison in a broad cohort of hospitals for relevant outcomes in a 'real life' setting outside of trial-modified situations. As such, it provides some insights into the triggers and consequences of the process of translating research into practice. We believe that the information we obtained may provide a perspective on the possible applicability and generalisability of clinical research in general and of research on the MET/RRS in particular.

Despite the above features, the study is retrospective and observational, with all of the associated limitations. We are able to comment on the uptake of MET services until April 2005 only and cannot assess the effect of the publication of the MERIT study [10] (published June 2005), which failed to show a beneficial effect of METs, on the subsequent introduction or possible removal of MET services. In addition, the MET status is known for only three quarters of the 172 ICU-equipped 'non-MERIT' hospitals in ANZ. It is possible that, if the missing 25% provided information, our findings would be altered. We were able to study only 29 hospitals, a relatively small number of the overall initial cohort (Figure 1). Thus, our findings may not be widely applicable or fully representative.

The assessment of the possible effect of the MET service on ICU admissions due to ward CAs and unplanned ICU admissions is also from a retrospective and before-and-after design, not from a randomised controlled trial. As such, it describes association and not causality. The reduction in ward CAs could be demonstrated only for patients subsequently admitted to the ICU. Therefore, we are unable to comment on the number of CAs that occurred in hospital wards but did not lead to ICU admission. These CAs may have increased or more people may have died from them, thus artificially reducing the number of patients admitted to the ICU. Furthermore, we observed that the introduction of the MET service was not associated with a reduction in the incidence of ICU readmissions or the mortality from admissions due to either ward CAs or ICU readmission. In addition, our study does not report on the incidence of other outcomes such as unexpected deaths or unplanned ICU admissions, which were used as the major outcomes for the MERIT study [10]. The ANZICS-APD does not collect information on these outcomes. Finally, the small cohort of hospitals that did not introduce an MET service was not sufficiently matched to provide a control and cannot be used as such. They simply provide illustrative data on the incidence of study outcomes over a contemporaneous 24-month period within the same health care systems. Nonetheless, these hospitals, like the non-MET hospitals in the MERIT study [10], showed a similar decrease in the incidence and rate of CAs admitted to the ICU from the ward. Finally, once we studied those hospitals from the MERIT study for which sufficient data were available, our findings were confirmed.

## Conclusion

Approximately 60% of ICU-equipped hospitals in ANZ report having introduced an MET service. In most of these hospitals, the service commenced prior to the publication of literature demonstrating the possible effectiveness of the MET on patient outcomes. In the 24 hospitals for which before-and-after data were available, introduction of an MET service was associated with no effect on the incidence of ICU readmissions, their mortality, or the mortality of patients admitted to the ICU after a ward CA. However, it was associated with a significant reduction in the incidence and rate of ICU admissions due to ward CAs. A similar reduction was also seen over a similar period of time among hospitals that had not introduced an MET service and in a cohort of hospitals that had participated in the MERIT study.

## Key messages

• A majority of hospitals in Australia and New Zealand (ANZ) appear to have introduced a Medical Emergency Team (MET) system.

• The introduction of such systems in ANZ occurred mostly before any publications reporting the possible effectiveness of such systems.

• The introduction of MET systems in ANZ appeared to be a response to publications highlighting the incidence of adverse events in hospitals.

• The introduction of MET systems in ANZ was associated (in hospitals for which data were available) with a temporal trend toward reduced intensive care unit admissions secondary to a ward cardiac arrest. However, a similar trend was seen in hospitals that did not have an MET system.

## Abbreviations

ANZ = Australia and New Zealand; ANZICS = Australian and New Zealand Intensive Care Society; ANZICS-APD = Australian and New Zealand Intensive Care Society Adult Patient Database; APD = Adult Patient Database; ARCCCR = Australian and New Zealand Intensive Care Society Research Centre for Critical Care Resources; CA = cardiac arrest; ICU = intensive care unit; IHI = Institute of Health Improvement; MERIT = Medical Emergency Response and Intervention Trial; MET = Medical Emergency Team; RRS = Rapid Response System.

## Competing interests

The authors declare that they have no competing interests.

## Authors' contributions

DJ and RB designed, executed, and wrote up the study. CG, GKH, and JM obtained the study data and assisted with study development and execution. All authors read and approved the final manuscript.

## References

[B1] Franklin C, Mathew J (1994). Developing strategies to prevent in hospital cardiac arrest: analysing responses of physicians and nurses in the hours before the event. Crit Care Med.

[B2] Kause J, Smith G, Prytherch D, Parr M, Flabouris A, Hillman K (2004). A comparison of antecedents to cardiac arrests, deaths and emergency intensive care admissions in Australia and New Zealand, and the United Kingdom – the ACADEMIA study. Resuscitation.

[B3] Schein RM, Hazday N, Pena M, Ruben BH, Sprung CL (1990). Clinical antecedents to in-hospital cardiopulmonary arrest. Chest.

[B4] Lee A, Bishop G, Hillman KM, Daffurn K (1995). The Medical Emergency Team. Anaesth Intensive Care.

[B5] Bellomo R, Goldsmith D, Uchino S, Buckmaster J, Hart GK, Opdam H, Silvester W, Doolan L, Gutteridge G (2003). A prospective before-and-after trial of a medical emergency team. Med J Aust.

[B6] Buist MD, Moore GE, Bernard SA, Waxman BP, Anderson JN, Nguyen TV (2002). Effects of a medical emergency team on reduction of incidence of and mortality from unexpected cardiac arrests in hospital: preliminary study. BMJ.

[B7] DeVita M (2005). Medical emergency teams: deciphering clues to crises in hospitals. Crit Care.

[B8] Goldhill DR, Worthington L, Mulcahy A, Tarling M, Sumner A (1999). The patient-at-risk team: identifying and managing seriously ill ward patients. Anaesthesia.

[B9] Ball C, Kirkby M, Williams S (2003). Effect of the critical care outreach team on patient survival to discharge from hospital and readmission to critical care: non-randomised population based study. BMJ.

[B10] Hillman K, Chen J, Cretikos M, Bellomo R, Brown D, Doig G, Finfer S, Flabouris A (2005). MERIT study investigators: Introduction of the medical emergency team (MET) system: a cluster-randomised controlled trial. Lancet.

[B11] Protecting 5 million lives from harm. http://www.ihi.org/IHI/Topics/CriticalCare/IntensiveCare/Changes/EstablishaRapidResponseTeam.htm.

[B12] Stow PJ, Hart GK, Higlett T, George C, Herkes R, McWilliam D, Bellomo R, for the ANZICS Database Management Committee (2006). Development and implementation of a high-quality clinical database: the Australian and New Zealand Intensive Care Society Adult Patient Database. J Crit Care.

[B13] Bristow PJ, Hillman KM, Chey T, Daffurn K, Jacques TC, Norman SL, Bishop GF, Simmons EG (2000). Rates of in-hospital arrests, deaths and intensive care admissions: the effect of a medical emergency team. Med J Aust.

[B14] Buist MD, Jarmolowski E, Burton PR, Bernard SA, Waxman BP, Anderson J (1999). Recognising clinical instability in hospital patients before cardiac arrest or unplanned admission to intensive care. A pilot study in a tertiary-care hospital. Med J Aust.

[B15] Goldhill DR, White SA, Sumner A (1999). Physiological values and procedures in the 24 h before ICU admission from the ward. Anaesthesia.

[B16] McQuillan P, Pilkington S, Allan A, Taylor B, Short A, Morgan G, Nielsen M, Barrett D, Smith G, Collins CH (1998). Confidential inquiry into quality of care before admission to intensive care. BMJ.

[B17] Smith AF, Wood J (1998). Can some in-hospital cardio-respiratory arrests be prevented? A prospective survey. Resuscitation.

[B18] Wilson RM, Runciman WB, Gibberd RW, Harrison BT, Hamilton JD (1995). The quality in Australian health care study. Med J Aust.

[B19] Overview of the 100,000 Lives Campaign. http://www.ihi.org/IHI/Programs/Campaign/100kCampaignOverviewArchive.htm.

[B20] Rapid Response Teams: The Case for Early Intervention. http://www.ihi.org/IHI/Topics/CriticalCare/IntensiveCare/ImprovementStories/RapidResponseTeamsTheCaseforEarlyIntervention.htm.

[B21] Hodgetts JJ, Kenward G, Vlackonikolis I (2002). Incidence, location and reasons for avoidable in-hospital cardiac arrest in a district general hospital. Resuscitation.

[B22] Foraida MI, DeVita MA, Braithwaite RS (2003). Improving the utilization of medical crisis teams (Condition C) at an urban tertiary care hospital. J Crit Care.

[B23] Bellomo R, Goldsmith D, Uchino S (2004). Prospective controlled trial of effect of medical emergency team on postoperative morbidity and mortality rates. Crit Care Med.

